# Supplementary material for: Improving drug identification in overdose death surveillance by using clinical natural language processing models
Source: J Forensic Sci. Author manuscript; Available in PMC 2026 May 8. (PMC13139796; doi:10.1111/1556-4029.70281)
Supplement: Table S1 - S5 [file NIHMS2170629-supplement-Table_S1_-_S5.docx]

**SUPPLEMENTAL MATERIALS**

TABLE S1 Full list of large language models (LLMs) tested and their macro-average F1 scores.

| **Model** | **Finetuned** | **0-shot** | **3-shot** | **5-shot** | **10-shot** |
| --- | --- | --- | --- | --- | --- |
| Llama-3.2-1B-Instruct | No | 0.261 | 0.205 | 0.242 | 0.277 |
| Llama-3.2-1B-Instruct | Yes | 0.951 | 0.739 | 0.645 | 0.661 |
| Llama-3.2-3B-Instruct | No | 0.819 | 0.849 | 0.806 | 0.808 |
| Llama-3.2-3B-Instruct | Yes | 0.959 | 0.844 | 0.787 | 0.757 |
| Llama-3.1-8B-Instruct | No | 0.924 | 0.888 | 0.883 | 0.878 |
| Llama-3.1-8B-Instruct | Yes | 0.959 | 0.932 | 0.926 | 0.921 |
| Qwen3-0.6B | No | 0.447 | 0.469 | 0.498 | 0.514 |
| Qwen3-0.6B | Yes | 0.916 | 0.899 | 0.871 | 0.866 |
| Qwen3-1.7B | No | 0.731 | 0.79 | 0.795 | 0.796 |
| Qwen3-1.7B | Yes | 0.959 | 0.968 | 0.965 | 0.964 |
| Qwen3-4B | No | 0.938 | 0.92 | 0.928 | 0.886 |
| Qwen3-4B | Yes | 0.962 | 0.961 | 0.958 | 0.963 |
| Qwen3-8B | No | 0.944 | 0.936 | 0.939 | 0.942 |
| Qwen3-8B | Yes | 0.946 | 0.939 | 0.943 | 0.942 |

TABLE S2 Error analysis and reasoning on internal dataset.

| **Drug Class** | **FP** | **FN** | **Total Errors** | **Possible Reasons** |
| --- | --- | --- | --- | --- |
| Methamphetamine | 1 | 0 | 1 | Occasionally detects MDMA as methamphetamine due to shared naming |
| Heroin | 1 | 0 | 1 | Associating other drugs with combined heroin use as well as cardiovascular related wording |
| Cocaine | 0 | 0 | 0 | N/A |
| Fentanyl | 0 | 0 | 0 | N/A |
| Alcohol | 2 | 0 | 2 | Associating “toxicity” or the combination of many drugs with alcohol |
| Prescription Opioids | 0 | 0 | 0 | N/A |
| Any Opioids | 0 | 1 | 1 | Unable to detect “ISOTONITAZENE” as an opioid |
| Benzodiazepines | 2 | 1 | 3 | Detecting a benzothiazepine as a benzodiazepine, unable to detect some benzos |
| Others | 3 | 0 | 3 | Overly sensitive especially when many different drugs are mentioned, mostly this occurs when there are lots of benzos also present |
| Any Drugs | 0 | 0 | 0 | N/A |
| **Total** | 9 | 2 | 11 | N/A |

TABLE S3 Error analysis and reasoning on external dataset.

| **Drug Class** | **FP** | **FN** | **Total Errors** | **Possible Reasons** |
| --- | --- | --- | --- | --- |
| Methamphetamine | 6 | 2 | 8 | Detecting MDMA as methamphetamine when written in full |
| Heroin | 4 | 0 | 4 | Confusion arises when many different substances mentioned, heroin is predicted although not in the substances |
| Cocaine | 4 | 2 | 6 | Associates drug use and cardiovascular use with cocaine rarely classifies this as cocaine use |
| Fentanyl | 0 | 0 | 0 | N/A |
| Alcohol | 0 | 6 | 6 | Misses mentions of cocaethylene, where alcohol or ethanol is not specifically mentioned |
| Prescription opioids | 0 | 0 | 0 | N/A |
| Any Opioids | 0 | 3 | 3 | Missed mentions of mitragynine as an opioid |
| Benzodiazepines | 4 | 7 | 11 | Misclassified benzothiazepines as benzodiazepines and missed some generic mention of benzodiazepines |
| Others | 12 | 72 | 84 | Overly sensitive when many different drugs are present, in the false negative case the model misses acetaminophen and some antihistamines |
| Any Drugs | 3 | 21 | 24 | Mention of drug use without specific mention of which drugs causes the model to miss these cases |
| Total | 33 | 113 | 146 | N/A |

TABLE S4 Metrics describing the performance of the single label classifiers, comparison of different embedding methods when paired with different model architectures.

|  |  | **BioClinicalBERT**  **embeddings** | **GloVe Embeddings** | **CUI2Vec Embeddings** |
| --- | --- | --- | --- | --- |
| **Substance** | **Model** | **F-Score** | **F-Score** | **F-Score** |
| Any Opioid | SVM | 0.986 | 0.989 | 0.992 |
|  | XGBoost | 0.963 | 0.983 | 0.990 |
|  | Random Forest | 0.949 | 0.970 | 0.986 |
|  | Logistic Regression | 0.984 | 0.988 | 0.991 |
| Heroin | SVM | 0.984 | 0.988 | 1.000 |
|  | XGBoost | 0.884 | 0.960 | 0.988 |
|  | Random Forest | 0.773 | 0.896 | 0.962 |
|  | Logistic Regression | 0.957 | 0.976 | 0.993 |
| Fentanyl | SVM | 0.998 | 0.999 | 0.999 |
|  | XGBoost | 0.965 | 0.989 | 0.998 |
|  | Random Forest | 0.942 | 0.973 | 0.997 |
|  | Logistic Regression | 0.993 | 0.997 | 0.998 |
| Prescription Opioid | SVM | 0.916 | 0.944 | 0.991 |
|  | XGBoost | 0.782 | 0.904 | 0.950 |
|  | Random Forest | 0.679 | 0.808 | 0.896 |
|  | Logistic Regression | 0.868 | 0.913 | 0.961 |
| Methamphetamine | SVM | 0.980 | 0.981 | 0.998 |
|  | XGBoost | 0.795 | 0.945 | 0.959 |
|  | Random Forest | 0.809 | 0.929 | 0.963 |
|  | Logistic Regression | 0.972 | 0.975 | 0.976 |
| Cocaine | SVM | 0.992 | 0.987 | 0.995 |
|  | XGBoost | 0.818 | 0.954 | 0.982 |
|  | Random Forest | 0.807 | 0.890 | 0.955 |
|  | Logistic Regression | 0.981 | 0.973 | 0.987 |
| Benzodiazepine | SVM | 0.949 | 0.752 | 0.752 |
|  | XGBoost | 0.790 | 0.862 | 0.679 |
|  | Random Forest | 0.697 | 0.733 | 0.672 |
|  | Logistic Regression | 0.895 | 0.723 | 0.743 |
| Alcohol | SVM | 0.968 | 0.945 | 0.915 |
|  | XGBoost | 0.885 | 0.954 | 0.914 |
|  | Random Forest | 0.760 | 0.949 | 0.917 |
|  | Logistic Regression | 0.962 | 0.939 | 0.914 |
| Others | SVM | 0.843 | 0.767 | 0.805 |
|  | XGBoost | 0.714 | 0.834 | 0.763 |
|  | Random Forest | 0.721 | 0.760 | 0.775 |
|  | Logistic Regression | 0.841 | 0.755 | 0.776 |
| Abbreviations: BERT, Bidirectional Encoder Representations from Transformers; CUI, Concept Unique Identifiers; GloVe, Global Word Embedding Vectors; SVM, Support Vector Machine; XGBoost, Extreme Gradient Boosting | | | | |

TABLE S5 Test Results Single Label Means with 95% Confidence Intervals (best model found on validation set: Support Vector Machine (SVM)).

|  | **Mean (95% Confidence Interval)** | | | | | | | | |
| --- | --- | --- | --- | --- | --- | --- | --- | --- | --- |
| **Metric** | **Any opioid** | **Heroin** | **Fentanyl** | **Prescription Opioids** | **Meth** | **Cocaine** | **Benzodiazepines** | **Alcohol** | **Others** |
| F-Score | 0.982  (0.982-0.983) | 0.962  (0.961-0.963) | 0.997  (0.996-0.997) | 0.840  (0.837-0.843) | 0.953  (0.952-0.955) | 0.976  (0.975-0.977) | 0.892  (0.890-0.895) | 0.934  (0.933-0.935) | 0.773  (0.769-0.776) |
| AUROC | 0.999  (0.999-1.00) | 0.998  (0.997-0.998) | 1.000  (1.000-1.000) | 0.996  (0.996-0.996) | 1.000  (1.000-1.000) | 0.998  (0.998-0.998) | 0.997  (0.997-0.997) | 0.999  (0.999-0.999) | 0.992  (0.992-0.993) |
| AUPRC | 0.998  (0.998-0.998) | 0.984  (0.983-0.985) | 1.000  (1.000-1.000) | 0.942  (0.940-0.944) | 0.995  (0.994-0.995) | 0.992  (0.991-0.992) | 0.958  (0.956-0.960) | 0.992  (0.991-0.992) | 0.875  (0.872-0.879) |
| Abbreviations: AUROC, area under the receiver operator curve; AUPRC, area under the precision recall curve | | | | | | | | | |
